# Meperidine and skin surface warming additively reduce the shivering threshold: a volunteer study

**DOI:** 10.1186/cc5709

**Published:** 2007-02-23

**Authors:** Oliver Kimberger, Syed Z Ali, Monica Markstaller, Sandra Zmoos, Rolf Lauber, Corinne Hunkeler, Andrea Kurz

**Affiliations:** 1 Department of Anaesthesiology, University of Bern, CH-3010 Bern, Switzerland; 2 Outcomes Research Institute, University of Louisville, 2301 S 3RD St, Louisville, KY 40292-2001, USA

## Abstract

**Introduction:**

Mild therapeutic hypothermia has been shown to improve outcome for patients after cardiac arrest and may be beneficial for ischaemic stroke and myocardial ischaemia patients. However, in the awake patient, even a small decrease of core temperature provokes vigorous autonomic reactions–vasoconstriction and shivering–which both inhibit efficient core cooling. Meperidine and skin warming each linearly lower vasoconstriction and shivering thresholds. We tested whether a combination of skin warming and a medium dose of meperidine additively would reduce the shivering threshold to below 34°C without producing significant sedation or respiratory depression.

**Methods:**

Eight healthy volunteers participated on four study days: (1) control, (2) skin warming (with forced air and warming mattress), (3) meperidine (target plasma level: 0.9 μg/ml), and (4) skin warming plus meperidine (target plasma level: 0.9 μg/ml). Volunteers were cooled with 4°C cold Ringer lactate infused over a central venous catheter (rate ≈ 2.4°C/hour core temperature drop). Shivering threshold was identified by an increase of oxygen consumption (+20% of baseline). Sedation was assessed with the Observer's Assessment of Alertness/Sedation scale.

**Results:**

Control shivering threshold was 35.5°C ± 0.2°C. Skin warming reduced the shivering threshold to 34.9°C ± 0.5°C (*p *= 0.01). Meperidine reduced the shivering threshold to 34.2°C ± 0.3°C (*p *< 0.01). The combination of meperidine and skin warming reduced the shivering threshold to 33.8°C ± 0.2°C (*p *< 0.01). There were no synergistic or antagonistic effects of meperidine and skin warming (*p *= 0.59). Only very mild sedation occurred on meperidine days.

**Conclusion:**

A combination of meperidine and skin surface warming reduced the shivering threshold to 33.8°C ± 0.2°C via an additive interaction and produced only very mild sedation and no respiratory toxicity.

## Introduction

Considerable evidence in animals indicates that mild hypothermia provides substantial protection against cerebral [1-4] and myocardial [5,6] ischaemia. In humans, mild hypothermia has been shown to improve neurologic outcome and to reduce mortality after cardiac arrest [7,8].

Mild hypothermia with core temperatures between 33°C and 34°C is relatively easy to induce during general anaesthesia because anaesthetics profoundly impair thermoregulatory responses [9-12]. However, induction of mild hypothermia in awake patients after acute myocardial infarction or acute ischaemic stroke may be difficult. Minute reductions (> 0.2°C) in core temperature trigger aggressive autonomic reactions–namely, arteriovenous shunt vasoconstriction and shivering–which *per se *impair efficient core cooling. Consequently, to lower the vasoconstriction and shivering thresholds, numerous studies have searched for drugs and drug combinations, including meperidine [13], buspirone [14], dexmedetomidine [15], clonidine [16], nefopam [17], and several others. Furthermore, non-pharmacological treatments–arm and face warming [18] or whole-body skin warming[19]–have been investigated with regard to their impact on thermoregulatory thresholds.

Meperidine, in contrast to other opioids, possesses additional anti-shivering action at equianalgesic doses and inhibits shivering twice as much as vasoconstriction [13,20]. However, when meperidine is used as a single drug, large plasma concentrations are needed to reduce the shivering threshold to below 34°C. These high plasma concentrations can cause significant respiratory depression and sedation and mandate close patient monitoring in an intensive care environment. They are thus not ideal in patient populations in which continuous evaluation of neurologic functioning is a major diagnostic and treatment approach.

In contrast, skin warming [19,21] efficiently lowers thermoregulatory thresholds without causing side effects. The skin contributes approximately 20% to the control of each thermoregulatory threshold [19,22], but skin surface warming alone is not sufficient to allow induction of mild hypothermia (33°C to 34°C) with catheter or intravenous fluid cooling. However, a combination of skin surface warming and a medium dose of meperidine (≈ 0.9 μg/ml plasma level) might additively decrease the shivering threshold to below 34°C. We therefore tested the hypothesis that the combination of meperidine and skin surface warming decreases the shivering threshold to below 34°C via an additive interaction without causing significant sedation or respiratory toxicity.

## Materials and methods

After obtaining approval by the Institutional Review Board of the University Hospital of Bern and written informed consent, we studied eight healthy volunteers on four days. Morphometric characteristics were age of 23 ± 2 years, weight of 68.6 ± 9.4 kg, height of 177 ± 7.5 cm, and body mass index of 22 ± 2. The volunteers fasted eight hours before the experiment. During treatment, they were minimally clothed and rested in a supine position on a standard patient bed equipped with a water mattress.

### Treatment protocol

All experiments were started between 8:30 and 9:00 a.m. to avoid any interference with thermoregulatory circadian fluctuations. After arrival in the volunteer laboratory, volunteers were randomly assigned to one of the four study days by means of a computer-generated randomisation list:

1. Control day: Placebo infusion, no skin warming.

2. Skin warming: The skin surface was warmed with a forced-air warming system (Bair Hugger; Arizant Inc., Eden Prairie, MN, USA) and a circulating water mattress (Medi-Therm III; Gaymar Industries, Inc., Orchard Park, NY, USA) set at maximum temperature (43°C and 41°C, respectively). Subsequently mean skin temperature was maintained at ≈ 35.8°C throughout the study.

3. Meperidine (target plasma level: 0.9 μg/ml): Meperidine was administered intravenously using an infusion regimen based on previously published pharmacokinetic data [23,24].

4. Meperidine (target plasma level: 0.9 μg/ml) plus skin warming (forced air plus water mattress) as described above.

A central venous catheter was inserted in the volunteer's left antecubital vein and used for blood sampling and infusion of cold Ringer lactate. For meperidine administration, an intravenous catheter was inserted into the right antecubital vein.

Once stable meperidine plasma levels and/or stable mean skin temperature was established on each study day and active arteriovenous shunt vasodilation was confirmed, lactated Ringer solution cooled to ≈ 4°C was infused on all study days at rates sufficient to decrease tympanic membrane temperature ≈ 2.4°C/hour (that is, ≈ 0.2°C every five minutes). The core temperature cooling rate was restricted to less than 2.5°C/hour because this rate was unlikely to trigger any dynamic thermoregulatory responses [25]. Fluid was given until the shivering threshold was identified or a total of up to 80 ml/kg of fluid was administered. Each study day ended upon detection of shivering. Subsequently, the volunteers were warmed until they were comfortable and had recovered from meperidine administration. Volunteers had an interval of at least 48 hours between study days; before the start of treatment, a blood sample was drawn to ensure that all meperidine remaining from previous study days had been eliminated.

### Measurements

Heart rate was measured continuously by means of an electrocardiogram, and blood pressure was determined using a non-invasive method at 10 minute intervals at the left arm. Core temperature was recorded from the tympanic membrane by means of Mon-a-Therm^® ^thermocouples (Mallinckrodt, Hazelwood, MO, USA). The tympanic probe was inserted by the volunteers until they felt the thermocouple touch the tympanic membrane; appropriate placement was confirmed when volunteers detected gentle rubbing of the attached wire. Additionally, tympanic probes were inserted into both ears, and a difference of less than or equal to 0.2°C confirmed correct placement. The aural canal was occluded with cotton, the probe was securely taped in place, and a gauze bandage was positioned over the external ear. Mean skin surface temperature was determined from 15 area-weighted sites [26].

All temperatures were recorded from thermocouples connected to calibrated Iso-Thermex^® ^16 channel electronic thermometers (Columbus Instruments, Columbus, OH, USA) that have an accuracy of 0.1°C and a precision of 0.01°C. Right index fingertip blood flow was quantified using volume plethysmography [27]. All measures of flow were recorded at five-minute intervals. Vasoconstriction threshold was defined by the core temperature triggeringa fingertip flow of ≈ 1 ml/minute, which roughly corresponds to a forearm-minus-fingertip skin temperature gradient near 0°C and indicates mild vasoconstriction [27].

Oxygen consumption was measured by a Vmax™ 29 n metabolic monitor (SensorMedics Corporation, Yorba Linda, CA, USA). Measurements were recorded every minute. A sustained increase in oxygen consumption of 20% above baseline identified the shivering threshold. The shivering and vasoconstriction thresholds were determined *post hoc *by an investigator blinded to treatment and core temperature.

As in previous studies, the level of sedation was assessed using the Observer's Assessment of Alertness/Sedation (OAA/S) scale [28] (Table 1). Thermal comfort was evaluated at 10-minute intervals with a 100 mm-long visual analogue scale. Zero millimetres was defined as the worst imaginable cold, 50 mm as thermal comfort, and 100 mm as the worst imaginable heat. Both scores were obtained at 10-minute intervals throughout cooling. Venous blood was sampled at three time points: before the start of meperidine infusion (to ensure that no meperidine plasma levels remained from previous study days) and at each thermoregulatory threshold for measurement of meperidine plasma concentrations.

**Table 1:** Observer's Assessment of Alertness/Sedation scale

Sub-score	Responsiveness	Speech	Facial expression	Eyes
5	Responds readily to name spoken in normal tone	Normal	Normal	Clear, no ptosis
4	Lethargic response to name spoken in normal tone	Mild slowing or thickening	Mild relaxation	Glazed or mild ptosis
3	Responds only after name is spoken loudly and/or repeatedly	Slurring or prominent slowing	Marked relaxation	Glazed and marked ptosis
2	Responds only after mild prodding or shaking	Few recognised words		
1	Does not respond to mild prodding or shaking			

The final score is the sum of the Responsiveness, Speech, Facial expression, and Eyes component scores. Thus, a 'wide awake' score = 20 and a 'deeply sedated' score = 9.

### Meperidine plasma level analysis

Blood samples were centrifuged at 4°C (2,000 *g*, 30 minutes), and the supernatant plasma was transferred to glass vials with a screw cap and stored at -20°C until analysis. Prior to extraction, plasma samples were allowed to thaw at room temperature. To 0.2 ml of plasma, 200 ng of piperidolate (Sigma-Aldrich, St. Louis, MO, USA) as internal standard (IS) and 0.05 ml 1 N sodium hydroxide solution were added and vortexed (5 seconds). Six tenths of a millilitre of a heptane-ethylacetate mixture (1:1, vol/vol) was added and vortexed for 30 seconds. The phases were separated by standing at room temperature for three minutes and centrifugation (1,300 *g*, three minutes). The water phase was flash-frozen in a 2-propanol dry ice bath. The organic phase was decanted into a second tube and dried under a gentle stream of nitrogen in a water bath (approximately 40°C). The residues were redissolved in 0.1 ml of extraction mixture, and 1 μl was injected splitless into the gaschromatograph (Agilent 6890; Agilent Technologies, Inc., Santa Clara, CA, USA) equipped with a mass selective detector. The capillary column was a VF-Xms (Varian, Inc., Palo Alto, CA, USA) with a length of 12 m, an inner diameter of 0.2 mm, and a film thickness of 0.33 μm. The carrier gas was helium at a flow rate of 1.0 ml/minute. Operating temperatures were 250°C for the injector, 280°C for the detector transfer line, and 100°C for the oven, which increased (30°C/minute) to 300°C and was held at that temperature for six minutes. The mass selective detector was operated in the electron impact mode (70 eV) with selected ion monitoring with a dwell time of 100 ms. The data were processed with proprietary mass spectrometer control software (HP G1701AA). The ions for quantitation were m/z 247 for meperidine and m/z 165 for the IS. Calibration curves with spiked and extracted plasma passed through the origin and were linear in the calibration range of 0.4 to 3.2 μg/ml, and correlation coefficients were more than 0.98. Coefficients of variation (CVs) of intra-day reproducibility (*n *= 5) were 3.1%, 3.2%, and 4.4% for meperidine in control samples containing 2.1, 1.3, and 0.7 μg/ml, respectively. Intra-day CVs were 3.5%, 3.6%, and 5.3% at the same concentrations, respectively. The limit of quantitation at a signal-to-noise ratio of 10:1 was 0.1 μg/ml. The recovery of meperidine and IS was more than 98%.

### Statistical analysis

Ambient temperature and physiological responses on each study day were first averaged for each volunteer; data obtained between the onset of shivering noticed by the observer and clinically relevant shivering (oxygen consumption +20%) were also included. The resulting values were then averaged among volunteers. Data were tested for normal distribution by means of Q-Q plot and Shapiro-Wilk test. All data were normally distributed. Results of the four study days and meperidine levels were compared using the Friedman test and the Student-Newman-Keuls test.

Interaction between the two treatments was evaluated using analysis of variance for repeated measurements [29]. A significant interaction (antagonism or synergism) was indicated by a statistically significant interaction term between the factors 'meperidine' and 'skin warming.' A non-significant interaction term indicated an additive effect. All results are expressed as means ± standard deviations; differences are considered statistically significant when *p *is less than 0.05. SPSS 11.0 (SPSS Inc., Chicago, IL, USA) was used for statistical analysis, and Graphpad Prism 4.0 (GraphPad Software, Inc., San Diego, CA, USA) was used for figures.

## Results

Physiological or ambient variables were comparable on the four study days (Table 2). All volunteers were vasodilated before the cold-fluid infusion was started. There was no difference for meperidine plasma levels between the meperidine day and the meperidine plus skin warming day (*p *= 0.42), and meperidine plasma levels remained stable throughout the experiments. Meperidine produced only mild sedation; OAA/S scale was not significantly decreased on the meperidine day (*p *= 0.10, versus control) or the meperidine plus skin warming day (*p *= 0.10, versus control) (Table 2).

**Table 2:** Confounding factors and results

	Control	Skin warming	Meperidine	Combined
Ambient temperature (°C)	22.6 ± 0.46	22.3 ± 0.5	22.1 ± 0.7	22.3 ± 0.7
Mean arterial pressure (mm Hg)	89.3 ± 7.1	87.9 ± 10.9	87.8 ± 9.4	88.6 ± 5.4
Heart rate (beats per minute)	65.3 ± 7.8	67.9 ± 11.7	62.5 ± 8.2	69.4 ± 9.6
SpO_2 _(percentage)	99.3 ± 0.8	99.1 ± 0.6	98.6 ± 1.0	98.6 ± 0.9
PCO_2 _(mm Hg)	5.1 ± 0.3	5.1 ± 0.5	5.0 ± 0.4	5.2 ± 0.8
Ringer lactate (ml/kg)	22.0 ± 4.5	50.6 ± 13.0^a^	58.4 ± 12.8^a^	84.3 ± 6.8^a^
Meperidine (μg/ml)	N/A	N/A	0.98 ± 0.20	0.96 ± 0.19
Thermal comfort VAS	26.9 ± 5.0	28.9 ± 9.3	34.8 ± 8.6^b^	43.6 ± 9.6^c^
OAA/S scale	20 ± 0	19.9 ± 0.3	19.5 ± 0.7	19.4 ± 0.7
Mean skin temperature start (°C)^d^	35.7 ± 0.4	35.8 ± 0.3	35.8 ± 0.2	35.8 ± 0.2
Mean skin temperature end (°C)^e^	34.3 ± 0.7	35.6 ± 0.3^a^	33.8 ± 0.6	35.4 ± 0.3^a^
Core temperature vasoconstriction (°C)	36.5 ± 0.2	36.1 ± 0.2^a^	35.8 ± 0.3^a^	35.6 ± 0.3^a^
Core temperature shivering (°C)	35.5 ± 0.2	34.9 ± 0.5^c^	34.2 ± 0.3^a^	33.8 ± 0.2^a^

^a^*p *< 0.01, ^b^*p *= 0.04, ^c^*p *= 0.01 (versus control). ^d^Mean skin temperature measured immediately before intravenous cooling. ^e^Mean skin temperature measured at the end of cooling. N/A, not applicable; OAA/S: Observer's Assessment of Alertness/Sedation; PCO_2_, partial pressure of carbon dioxide; SpO_2_, oxygen saturation as measured by pulse oximetry; VAS, visual analogue scale.

### Effect of meperidine and skin warming on the vasoconstriction threshold

Skin warming reduced the vasoconstriction threshold by -0.4°C ± 0.3°C (*p *< 0.01). Meperidine reduced the vasoconstriction threshold by -0.7°C ± 0.3°C (*p *< 0.01). The combination of meperidine and skin warming significantly reduced the vasoconstriction threshold by -0.9°C ± 0.2°C (*p *< 0.01; Table 2; all values compared to control day).

### Effect of meperidine and skin warming on shivering threshold

**Figure 1:**
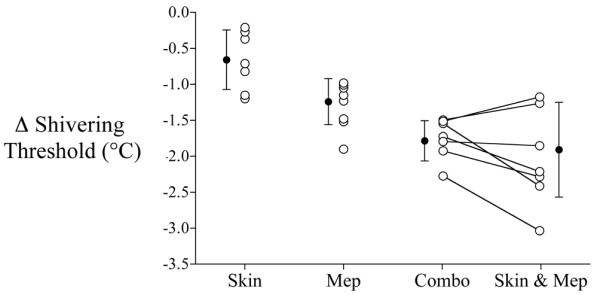
Reductions in the shivering threshold versus control. Bars indicate means ± standard deviation. Combo, actual effect of skin warming plus meperidine; Mep, meperidine; Skin, skin warming; Skin & Mep, calculated additive effect of skin warming plus meperidine.

Skin warming reduced the shivering threshold by -0.6°C ± 0.5°C (*p *= 0.01). Meperidine reduced the shivering threshold by -1.3°C ± 0.3°C (*p *< 0.01). The combination of meperidine and skin warming reduced the shivering threshold by -1.7°C ± 0.3°C (*p *< 0.01; Table 2; Figure 1; all values compared to control day).

There was no interaction between meperidine and skin warming for vasoconstriction (*p *= 0.20) or shivering (*p *= 0.59) thresholds. A combination of both treatments thus additively reduced the shivering threshold.

## Discussion

This study demonstrates that a combination of meperidine and skin surface warming additively reduces the shivering threshold to below 34°C and produces only very mild sedation and no respiratory toxicity. The combination of meperidine and skin surface warming can thus be recommended for facilitating the application of therapeutic mild hypothermia in the awake patient.

In humans, mild hypothermia for 24 hours has been shown to improve outcome after cardiac arrest [7,8] and the application of mild hypothermia has been advised by the International Liaison Committee on Resuscitation [30] and the European Resuscitation Council [31]. Currently, several clinical mild hypothermia studies in patients with ischaemic heart injury, brain trauma, or ischaemic stroke are ongoing.

In the majority of clinical mild hypothermia studies, general anaesthesia that patients received was often combined with generous amounts of muscle relaxants to suppress shivering. Although anaesthetics reduce shivering and vasoconstriction thresholds by 2°C to 3°C, these drugs are, unsurprisingly, not the first choice for induction of mild hypothermia in the awake patient; they cause significant sedation and respiratory depression and jeopardise airway patency.

Consequently, numerous studies have searched for other drugs or drug combinations that lower vasoconstriction and shivering thresholds. One of the most effective anti-shivering drugs has proven to be meperidine [32,33]. Large plasma concentrations of meperidine, when used as a single drug, are necessary to reduce shivering thresholds to below 34°C, which leads to sedation and respiratory depression. Smaller doses of meperidine in combination with other drugs (buspirone [14], dexmedetomidine [15], or magnesium [34]) have therefore been evaluated. The combination of meperidine and the anxiolytic drug buspirone has been the most effective. Mokhtarani and colleagues [14] demonstrated synergistic properties of this particular combination in contrast to other combinations. The combination of a relatively small dose of meperidine (0.3 ± 0.1 μg/ml) and a single dose of buspirone (30 mg orally) was able to lower the volunteers' shivering threshold to 33.4°C ± 0.7°C.

In the present study, we used meperidine in combination with skin surface warming and targeted a meperidine plasma level of 0.9 μg/ml. This combination (meperidine plus skin warming) has also been tested and used for the induction/maintenance of mild hypothermia with endovascular cooling in several clinical pilot studies in awake patients. Dixon and colleagues [35] used a combination of meperidine (75 to 100 mg loading dose, followed by 25 to 35 mg/hour continuously), skin warming, and oral buspirone (30 to 60 mg orally) to induce mild hypothermia (33.2°C core temperature) in 21 patients during coronary intervention for acute myocardial infarction. They reported good feasibility with mild shivering in nine patients. Leslie and colleagues [36] reported that a combination of a low level of meperidine (target plasma level: 0.4 mg/ml) and skin warming in 10 patients scheduled for neurosurgery was insufficient to prevent shivering. However, study results have also shown a large range of meperidine levels (0.1 to 0.9 μg/ml) and therefore have to be interpreted with caution. In a pilot study by De Georgia and colleagues [37], 18 ischaemic stroke patients were cooled to 33°C using a combination of meperidine (50 to 75 mg loading dose, followed by 25 to 35 mg/hour continuously), skin warming, and oral buspirone (60 mg). The hypothermia group had a significantly higher incidence of intubations compared to 22 normothermic patients. The authors stated that it was unclear whether severity of disease or drug-induced sedation caused the difference. In a recent feasibility study, Guluma and colleagues [38] used a combination of meperidine (loading dose 1 mg/kg, followed by 30 mg/hour continuously) and skin warming to induce core hypothermia (33.8°C) in 10 ischaemic stroke patients and reported satisfactory results.

However, only in the study by Leslie and colleagues [36] were meperidine levels measured and sedation assessed with a validated scale; in none of the aforementioned studies was the interaction between meperidine and skin warming examined. In contrast, by means of an approach described by Slinker [29], our present volunteer study is able to demonstrate an additive interaction between meperidine and skin warming.

Our study results are in accordance with those of previous studies. Using comparable levels of meperidine (≈ 1 μg/ml), Ikeda and colleagues [39] showed a decrease of the shivering threshold to 34.8°C ± 1.0°C (10 volunteers, cooling with intravenous cold fluid). The shivering threshold results on the meperidine day of our study are slightly lower (34.2°C ± 0.2°C), most likely because our volunteers had higher mean skin temperatures. In a skin warming study by Alfonsi and colleagues [21], forced-air warming decreased the shivering threshold of postoperative patients by ≈ -0.4°C (18 patients, forced-air warming). Our results show a slightly larger decrease of ≈ -0.6°C under comparable mean skin temperatures. Skin warming reduces oxygen consumption. Consequently, in volunteers on skin warming days, the threshold according to the present study's definition of shivering (+20% increase in oxygen consumption) is reached at a relatively lower core temperature, which may explain the abovementioned difference.

We used the combination of meperidine and skin warming in healthy volunteers only for induction of mild hypothermia. If our treatment had been applied for more than 24 hours, meperidine toxicity may well have become an important issue. Repeated doses of meperidine can induce feelings of shakiness, seizures, tremor, mood changes, and muscle weakness. Norpethidine, a meperidine metabolite, is most likely responsible [40] and correlates with the side effects of meperidine [41]. Patients with renal insufficiency are particularly affected because they tend to accumulate norpethidine faster [42]. Renal insufficiency therefore has to be considered a relative contraindication to anti-shivering drug combinations that include meperidine.

In the present study, core hypothermia was induced by intravenous infusion of cold fluid (Ringer lactate, 4°C). This method has also been used successfully for induction of hypothermia in several clinical studies in patients with various types of neurologic injuries [43] and in patients after cardiac arrest [44,45]. Direct core cooling with cold-fluid infusion or intravenous cooling catheters has several advantages over conventional surface cooling. It is faster because heat is removed directly from the core rather than being required to pass through peripheral tissues, which insulate the core [46]. Core cooling can easily be combined with simultaneous skin surface warming. Finally, external cooling can become more difficult once core temperature is below the vasoconstriction threshold; this issue does not apply to cold-fluid cooling. However, despite the surprisingly good tolerance of fluid cooling by stroke or cardiac arrest patients, cold-fluid cooling may be an option for induction of hypothermia only, and other techniques (for example, an intravenous cooling catheter) are necessary for maintenance of hypothermia. We restricted core cooling rates to a range of 1°C/hour to 2°C/hour as we had previously shown that similar rates of skin surface cooling do not directly affect the shivering threshold and are unlikely to produce dynamic thermoregulatory responses [25].

A limitation of our study is that it was conducted in young, healthy volunteers. Results from volunteer studies cannot always be extrapolated to clinical situations. It is not unlikely that meperidine-induced sedation–only very mild in healthy volunteers–may have a bigger impact on patients after cardiac arrest or stroke.

In the present study, we evaluated sedation with the validated OAA/S scale [28]. Although this scale does not detect subtle degrees of sedation, it is unlikely that these subtle changes are clinically relevant in the context of stroke and other life-threatening conditions. More relevant are respiratory depression and ensuing hypercapnia, which limit the administration of meperidine in typical ward settings. In our study, end-tidal PCO_2 _(partial pressure of carbon dioxide) did not increase and no signs of respiratory depression could be observed.

## Conclusion

A combination of meperidine and skin surface warming reduced the shivering threshold to 33.8°C ± 0.2°C via an additive interaction and produced only very mild sedation and no respiratory toxicity. This combination can therefore be considered a recommendable and feasible regimen for induction and maintenance of therapeutic mild hypothermia.

## Key messages

• Meperidine and skin surface warming additively reduced the shivering and vasoconstriction thresholds.

• A combination of meperidine (≈ 0.9 μg/ml plasma level) and skin surface warming lowered the shivering threshold to 33.8°C ± 0.2°C and allowed the induction of mild therapeutic hypothermia with only very mild sedation and no respiratory toxicity.

## Abbreviations

CV = coefficient of variation; IS = internal standard; OAA/S = Observer's Assessment of Alertness/Sedation.

## Competing interests

The authors declare that they have no competing interests.

## Authors' contributions

OK conceived the study, drafted the manuscript, performed the statistical analysis, and performed the volunteer experiments. SZA conceived the study and performed the volunteer experiments. SZ, CH and MM performed the volunteer experiments. RL performed the meperidine plasma level analysis. AK conceived the study and drafted the manuscript. All authors read and approved the final version of the manuscript.
